# Metabolism, morphology and transcriptome analysis of oscillatory behavior of *Clostridium butyricum* during long-term continuous fermentation for 1,3-propanediol production

**DOI:** 10.1186/s13068-020-01831-8

**Published:** 2020-11-25

**Authors:** Jin-Jie Zhou, Jun-Tao Shen, Xiao-Li Wang, Ya-Qin Sun, Zhi-Long Xiu

**Affiliations:** School of Bioengineering, Dalian University of Technology, Dalian, Liaoning 116024 People’s Republic of China

**Keywords:** *Clostridium butyricum*, Oscillatory behavior, 1,3-Propanediol, Glycerol metabolism, Morphology, Transcriptome

## Abstract

**Background:**

Oscillation is a special cell behavior in microorganisms during continuous fermentation, which poses threats to the output stability for industrial productions of biofuels and biochemicals. In previous study, a spontaneous oscillatory behavior was observed in *Clostridium butyricum-*intensive microbial consortium in continuous fermentation for 1,3-propanediol (1,3-PDO) production from glycerol, which led to the discovery of oscillation in species *C. butyricum*.

**Results:**

Spontaneous oscillations by *C. butyricum* tended to occur under glycerol-limited conditions at low dilution rates. At a glycerol feed concentration of 88 g/L and a dilution rate of 0.048 h^−1^, the oscillatory behavior of *C. butyricum* was observed after continuous operation for 146 h and was sustained for over 450 h with an average oscillation period of 51 h. During oscillations, microbial glycerol metabolism exhibited dramatic periodic changes, in which productions of lactate, formate and hydrogen significantly lagged behind that of other products including biomass, 1,3-PDO and butyrate. Analysis of extracellular oxidation–reduction potential and intracellular ratio of NAD^+^/NADH indicated that microbial cells experienced distinct redox changes during oscillations, from oxidized to reduced state with decreasing of growth rate. Meanwhile, *C. butyricum* S3 exhibited periodic morphological changes during oscillations, with aggregates, elongated shape, spores or cell debris at the trough of biomass production. Transcriptome analysis indicated that expression levels of multiple genes were up-regulated when microbial cells were undergoing stress, including that for pyruvate metabolism, conversion of acetyl-CoA to acetaldehyde as well as stress response.

**Conclusion:**

This study for the first time systematically investigated the oscillatory behavior of *C. butyricum* in aspect of occurrence condition, metabolism, morphology and transcriptome. Based on the experimental results, two hypotheses were put forward to explain the oscillatory behavior: disorder of pyruvate metabolism, and excessive accumulation of acetaldehyde.

## Background

Glycerol is the byproduct of biodiesel and oleochemical industries with worldwide oversupply and low price [1–3]. Bioconversion of such a waste material to value-added chemicals is an attractive option in consideration of both economic and environmental benefits. Glycerol can be converted to numerous chemicals such as 1,3-propanediol (1,3-PDO), 1,2-propanediol, ethanol, butanol, citrate and succinate by microbial fermentation [4–6]. Among these cases, 1,3-PDO is one of the most valuable products with versatile applications in medicine, cosmetic and textile industry. Specifically, 1,3-PDO is the building block for biosynthesis of polymers for example polytrimethylene terephthalate (PTT), which has a remarkable potential in plastic, fiber, textile and coatings industries [7, 8].

In nature, there are a few types of microorganisms that can directly convert glycerol to 1,3-PDO, including genus *Clostridium*, *Klebsiella*, *Lactobacillus*, *Enterobacter* and *Citrobacte*r [9]*.* Among these microorganisms, *Clostridium butyricum* is one of the best candidates owing to the competitive production titer, yield and productivity [10]. *C. butyricum* is a strictly anaerobic, Gram-positive and sporulating bacterium, which is widely distributed in soil, sludge as well as intestines of humans and animals [11]. Glycerol metabolism of *C. butyricum* can be divided into reductive and oxidative routes [12]. In the reductive pathway, *C. butyricum* first converts glycerol to 3-hydroxypropionaldehyde (3-HPA) via glycerol dehydratase (GDHt), followed by formation of 1,3-PDO via 1,3-propanediol oxidoreductase (PDOR) using NADH as the electron donor. Studies showed that glycerol dehydratase from *C. butyricum* is independent of high-cost coenzyme B12 [13], which makes this species another economical advantage than other B12-dependent 1,3-PDO producers such as *Klebsiella pneumoniae* [14], *Citrobacter freundii* [15] and *Clostridium pasteurianum* [16]. In the oxidative pathway, glycerol is first oxidized to dihydroxyacetone (DHA) via glycerol dehydrogenase (GDH), followed by production of pyruvate catalyzed by a series of enzymes. Next, pyruvate is converted either to lactate by lactate dehydrogenase (LDH) or to acetyl-CoA by pyruvate: ferredoxin oxidoreductase (PFO) and/or pyruvate: formate lyase (PFL). Acetyl-CoA can be ultimately converted to butyrate, acetate and ethanol. The oxidative pathway generates energy (ATP) and reducing power (NADH/NADPH) for microbial biosynthesis and 1,3-PDO production.

Continuous fermentation is a common operation mode in bulk biochemical industry with multiple advantages over batch and fed-batch operations, for example high product productivity and low operation costs [17, 18]. For 1,3-PDO production, to the best of our knowledge, the highest 1,3-PDO titers in continuous fermentation is 57.86 g/L using *C. butyricum*-intensive anaerobic consortium C2-2M and 50.77 g/L using pure culture *C. butyricum* S3 with competitive productivities, which approach the average level using fed-batch fermentation [9, 19]. However, despite the bright outlook for industrial application, performance instability of microorganisms during continuous fermentation is the key operational issue, which could be caused by internal reasons such as gene mutation and metabolism disorder, or external reasons such as microbial contamination [17, 18, 20, 21].

Oscillation is a ubiquitous behavior among prokaryotic and eukaryotic organisms. On one side, keeping periodic oscillation is critical for cell homeostasis in most eukaryotes in response to light–dark cycles, known as circadian rhythm. Perturbations of this oscillatory behavior would increase risk of metabolic disorders and diseases for humans [22, 23]. On the other side, in industrial production of biofuels and biochemicals, oscillatory behavior can cause unstable product output and put threat on process stability. Oscillation can occur spontaneously or be induced by perturbation of cultivation conditions, and period and amplitude of different oscillations are remarkably various. As for the mechanism, oscillatory behavior of a single organism mainly results from feedback interaction by environmental parameter or intracellular systems [24]. On one hand, in continuous cultivation, cell growth and metabolism would affect the environmental parameters such as pH, oxygen tension and concentration of extracellular metabolites, which in turn, would affect cell metabolism. Intracellular feedback regulation, on the other hand, includes cell cycle (rhythmic phenomena for higher animal/cell division for microorganism), induction/repression of enzyme and allosteric control of enzyme activity. For microorganism particularly, reasons for the oscillatory behavior are complex, which are associated with type of microorganisms and culture condition, and one species of microorganism could exist several potential mechanisms [25–28].

So far, many industrial microorganisms including *E. coli* [29], *Clostridium acetobutylicum* [30], *Saccharomyces cerevisiae* [31, 32], *Zymomonas mobilis* [33] and *Chlorella vulgaris* [34] have been found to have oscillatory behavior during continuous fermentation. When it comes to continuous 1,3-PDO production from glycerol, two microorganisms have been observed to have oscillatory behavior. *Klebsiella pneumoniae* showed sustained oscillation after dramatic environmental disturbance. Possible reasons for the oscillation could be pyruvate metabolism disorder [28] and/or toxicity of intermediate 3-HPA [27]. Recently, another 1,3-PDO producer, *Clostridium pasteurianum* exhibited spontaneous oscillation in continuous fermentation, probably caused by toxicity of byproduct butanol [26] and/or relative to concerted cycles of inhibition and activation of enzymes for glycerol oxidation [25]. Exploration of the microbial oscillation not only helps us to better understand the internal metabolic regulation of microbial cells, but also contributes to the process stability in industrial application.

In our previous study, a spontaneous oscillatory behavior was observed in continuous fermentation by *C. butyricum*-intensive microbial consortium under glycerol-limited conditions, which has been proved to be the metabolic feature of *C. butyricum* [19]. The present study systemically elaborated this newly found oscillatory behavior of *C. butyricum* in continuous fermentation, including occurrence conditions, metabolism, redox status as well as morphology. Furthermore, genome and transcriptome analyses were conducted to identify gene expression patterns during an oscillation cycle. Based on the existed experimental data, the significant features and possible reasons for the oscillatory behavior of *C. butyricum* S3 were discussed.

## Results

### Conditions for oscillation occurrence

In the previous study, the oscillatory behavior of *C. butyricum*-intensive microbial consortium C2-2M tended to occur in continuous fermentation under conditions when the residual glycerol concentrations were low, and no oscillation was observed in continuous fermentation when glycerol supply was sufficient for *C. butyricum* S3 [19]. It was suspected that the oscillatory behavior by *C. butyricum* S3, isolated from microbial consortium C2-2M would be observed under conditions with low residual glycerol concentrations as well. Therefore, continuous fermentations at different dilution rates and glycerol feed concentrations with low residual glycerol concentrations were scanned to identify the cultivation condition that would lead to oscillatory behavior for *C. butyricum* S3.

**Fig. 1:**
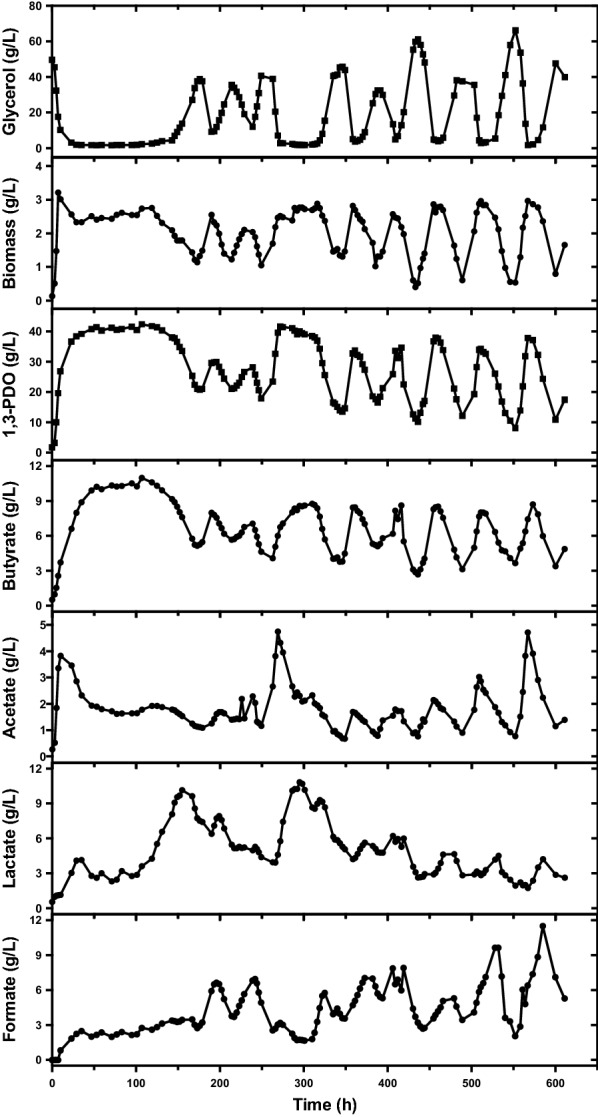
Continuous fermentation by *C. butyricum* S3 at a glycerol feed concentration of 88 g/L and a dilution rate of 0.048 h^−1^

**Fig. 2:**
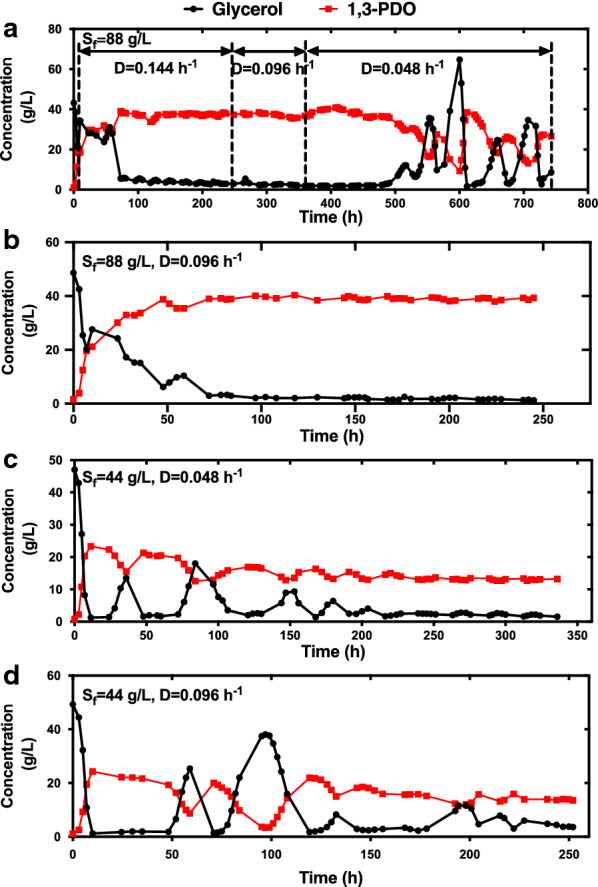
Glycerol consumption and 1,3-PDO production of *C. butyricum* S3 in continuous fermentations at **a** S_f_=88 g/L and stepwise decreasing D from 0.144 h^-1^ to 0.048 h^-1^, **b** S_f_=88 g/L and D=0.096 h^-1^, **c** S_f_=44 g/L and D=0.048 h^-1^, **d** S_f_=44 g/L and D=0.096 h^-1^. S_f_: glycerol feed concentration, D: dilution rate

As expected, in continuous fermentation at a glycerol feed concentration of 88 g/L and a dilution rate of 0.048 h^−1^, oscillation was observed after 146 h and sustained for over 450 h (Fig. 1), which was accordant with the oscillatory behavior by microbial consortium C2-2M [19]. More details about this oscillation would be discussed in the following sections. While at higher dilution rates of 0.144 and 0.096 h^−1^, no oscillations were detected at the same glycerol feed concentration after over 200 h (Fig. 2a, b, Additional file 1: Figs. S1, S2), despite most glycerol being consumed as well. Meanwhile, when stepwise decreasing the dilution rate from 0.144 to 0.096 h^−1^ then to 0.048 h^−1^, the oscillatory behavior was only observed after over 100 h of continuous operation at the lowest dilution rate of 0.048 h^−1^, accompanying with a significant metabolic shift from lactate to formate formation (Fig. 2a, Additional file 1: Fig. S1). The results indicated that the occurrence of oscillation by *C. butyricum* was tightly associated with operating conditions.

In addition, when decreasing the glycerol feed concentration to 44 g/L, the oscillatory phenomena of *C. butyricum* only occurred under at low dilution rates of 0.048 h^−1^ and 0.096 h^−1^ (Fig. 2c, d). However, the oscillatory behaviors in these two conditions were not able to sustain, but tended to damp out and vanished ultimately. The results indicated that besides requirement of glycerol-limited condition, operating at a comparable amount of feed glycerol (in this study, equal or higher than 88 g/L) was essential for *C. butyricum* S3 to maintain oscillation. In addition, the oscillations under these conditions occurred after shorter periods of steady state (13 and 38 h, respectively) than that under conditions at a glycerol feed concentration of 88 g/L and a dilution rate of 0.048 h^−1^. And after long-term cultivation, remarkable metabolic shift represented by significant accumulation of formate and decrease of lactate production was observed as well (Additional file 1: Figs. S3, S4).

In the following parts, continuous fermentation at a glycerol feed concentration of 88 g/L and a dilution rate of 0.048 h^−1^ was set as an example for exploration of the oscillatory behavior of *C. butyricum* S3, including metabolism, morphology and transcriptome.

### Metabolic profiles of *C. butyricum* S3 in typical oscillatory phases

As shown in Fig. 1, after feeding at late log phase (5 h), the culture rapidly consumed all glycerol after 23 h. During the period from 29 to 119 h, *C. butyricum* S3 exhibited a relatively stable metabolism. Nearly all glycerol was consumed, with average 1,3-PDO production of 40.66 ± 1.09 g/L. Butyrate was the main byproduct with average concentration of 10.02 ± 0.81 g/L. Concentrations of other byproducts including acetate, lactate and formate were maintained at low levels. Afterwards, during period from 119 to 146 h, although the residual glycerol concentration remained at a similarly low level as before, *C. butyricum* S3 exhibited a significant metabolic shift. The most apparent change was the lactate production, as the concentration increased more than twice from 4.26 to 9.07 g/L. Formate production also increased from 2.60 to 3.34 g/L. While productions of biomass, 1,3-PDO, butyrate and acetate were decreased to some extent.

After 146 h, the oscillation started and maintained for over 450 h with an average oscillation period of 51 h except from 249 to 346 h. From an overall viewpoint, the oscillatory behavior of *C. butyricum* became more regular at the later stage of fermentation after 450 h. During oscillations, glycerol consumption as well as productions of biomass, 1,3-PDO and butyrate displayed dramatic and regular fluctuations. While concentrations of lactate, formate and acetate exhibited less regular oscillations with inconsistent amplitudes. Specifically, lactate production was favored at early stage of oscillation and then was suppressed to low levels after 350 h, whereas significant increases in concentrations of formate and acetate were observed in the later period.

**Fig. 3:**
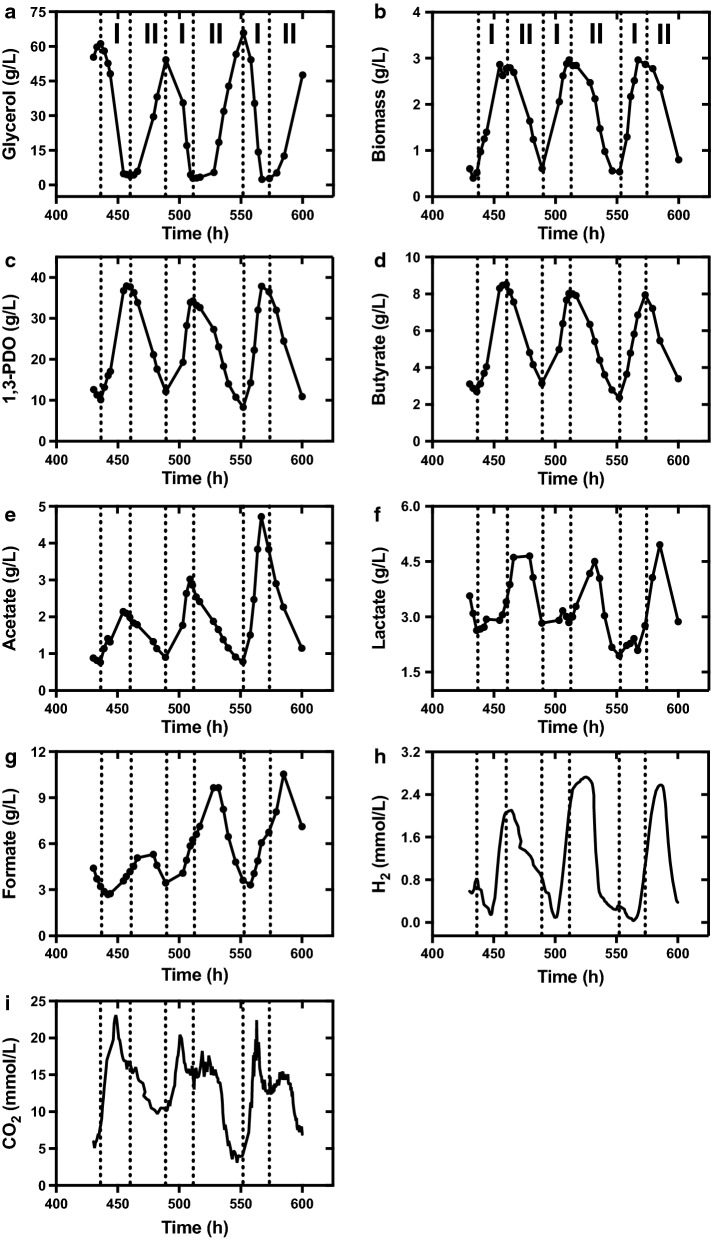
Oscillatory patterns of concentrations of glycerol (**a**), biomass (**b**), 1,3-PDO (**c**), butyrate (**d**), acetate (**e**), lactate (**f**), formate (**g**), H_2_ (**h**) and CO_2_ (**i**) in continuous fermentation at a glycerol feed concentration of 88 g/L and a dilution rate of 0.048 h^−1^

Changes in concentrations of substrate and products during three oscillation periods from 430 to 600 h were described in detail (Fig. 3). The oscillation cycle could be divided into the growth rising stage (stage I) and the falling stage (stage II). In general, stage I occupied less time than stage II (20–22 h vs. 32–41 h). During one oscillation cycle from stage I to stage II, glycerol concentration was dropped from around 60 g/L to around 3 g/L, and then gradually accumulated to the maximum value. Correspondingly, formation of microbial biomass, 1,3-PDO, butyrate and acetate first increased to the maximum values and then decreased back to the initial values. However, concentrations of lactate and formate showed significantly delayed oscillations compared with that of glycerol and other metabolites, in which the maximum values occurred at the middle of stage II, and minimum values occurred at early or middle of stage I.

Meanwhile, two main gases H_2_ and CO_2_ exhibited regular oscillations with similar frequencies. For H_2_ production, the fluctuation pattern was similar with that for lactate and formate, with the maximum values (2.47 ± 0.32 mmol/L) shown at middle of the stage II and the minimum values (0.07 ± 0.03 mmol/L) shown at the middle of the stage I. While CO_2_ accumulated ahead of all metabolites, with the maximum values of 21.98 ± 1.38 mmol/L shown at the middle of stage I. Afterwards, CO_2_ concentration started to decrease to some extent and then stayed steady for a while, followed by rapid decrease to the minimum value at the end of the stage II. It was worth attention that the time point when minimum level of H_2_ production occurred was corresponding to that when CO_2_ was accumulated to the maximum.

**Fig. 4:**
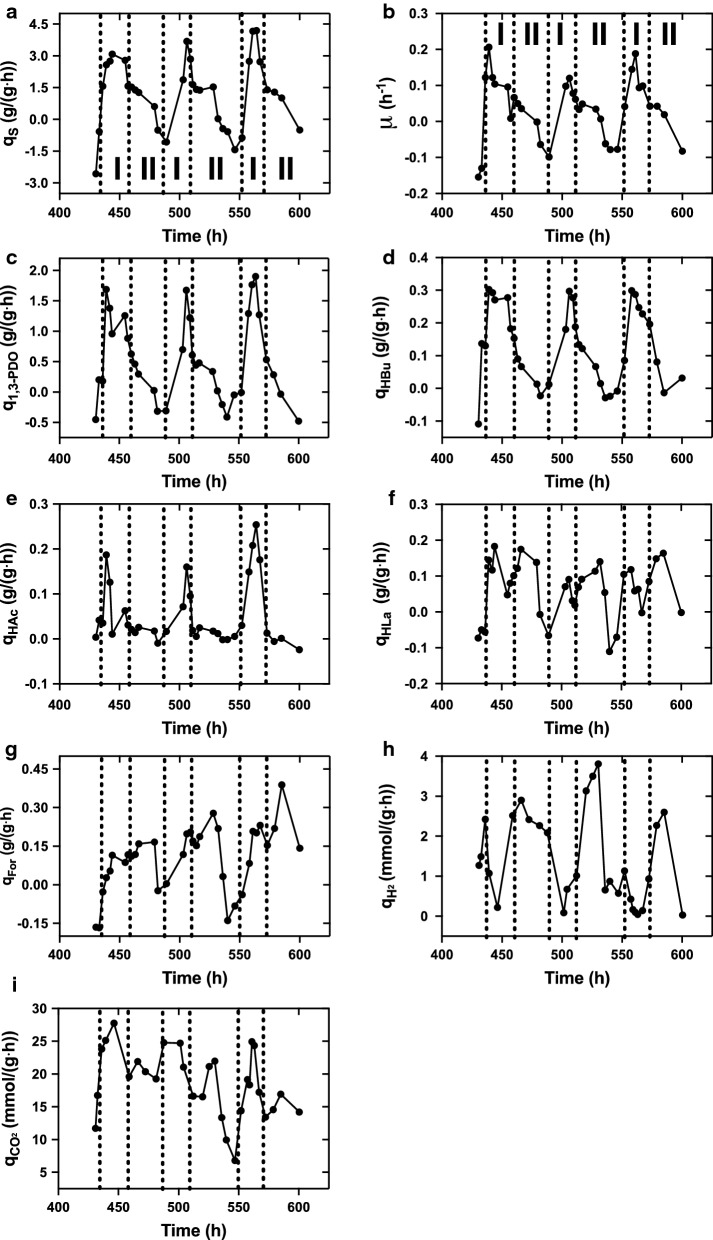
Oscillatory patterns of specific rates of glycerol consumption (**a**) and productions of biomass (**b**), 1,3-PDO (**c**), butyrate (**d**), acetate (**e**), lactate (**f**), formate (**g**), H_2_ (**h**) and CO_2_ (**i**)  in continuous fermentation at a glycerol feed concentration of 88 g/L and a dilution rate of 0.048 h^−1^

As kinetic analysis could reflect real-time metabolic flux, kinetic patterns of glycerol metabolism by *C. butyricum* were explored during the same oscillation periods (Fig. 4). The highest rates of glycerol consumption, biomass and 1,3-PDO productions occurred at the middle instead of the end of stage I. Butyrate and acetate showed consistent oscillation, while acetate production was inhibited more quickly and thoroughly than butyrate after achieving the maximum peak. The results indicated that microbial cells sensed the pressure before the macroscopic metabolic response during oscillations. After passing through the peak, rates of glycerol consumption and production of biomass, 1,3-PDO and butyrate were likely decreased into a short-term platform stages before completely falling to the minimum values.

However, specific production rates of lactate (q_HLa_) and formate (q_For_) showed different changing patterns. First, q_HLa_ showed consistently M shape during three periods of oscillation (430–600 h) with two peaks within one oscillation cycle: at the middle of stage I (444, 506 and 558 h) and at the middle of stage II (466, 532 and 579 h) (Fig. 4f). While the trough value occurred at late to end of both the stage I (454, 511 and 567 h) and stage II (489 and 540 h). Formate production rate showed similar but more irregular fluctuation. During the rising stage, q_For_ showed increasing tendency but slightly dropping. Afterwards, the rate further increased to the maximum value at the middle of the falling stage (479, 528 and 585 h), and finally drop to the minimum value at late of the falling stage (481 and 540 h). The period of the second increases was corresponding with the platform stage of the concentration metabolites.

For two gases, specific production rate of H_2_ lagged behind that of other liquid metabolites including 1,3-PDO and butyrate, in which the maximum value occurred at the end of stage I, and the minimum value of occurred at the late stage II or beginning of stage I. CO_2_ production rate showed similar pattern with that of lactate and formate, in which two peaks showed in one period of oscillation.

### Redox status

Besides metabolites, intracellular and extracellular redox states were monitored during oscillations (Additional file 1: Fig. S5). Intracellular redox status was determined by levels of intracellular redox cofactors NAD^+^/NADH. Intracellular NAD^+^ concentration showed similar oscillatory patterns with that of biomass and 1,3-PDO, whereas NADH remained at a low level during oscillations. As a result, periodic fluctuation of the ratio of NAD^+^/NADH indicated that cells were under more oxidative conditions in stage I than that in stage II. For extracellular redox status, the oxidation–reduction potential (ORP) showed a similar fluctuation pattern with the NAD^+^/NADH ratio, with the maximum ORP value of − 271 ± 72 mV at the middle of stage I and the minimum value of − 568 ± 11 mV at the end of stage I or slightly later. The consistent patterns of change in intracellular and extracellular redox states indicated that the redox status of the entire system including cells and cultural environment experienced dramatic and periodic fluctuation during oscillations, which shifted from oxidative to reductive conditions from stage I to stage II.

### Morphology

**Fig. 5:**
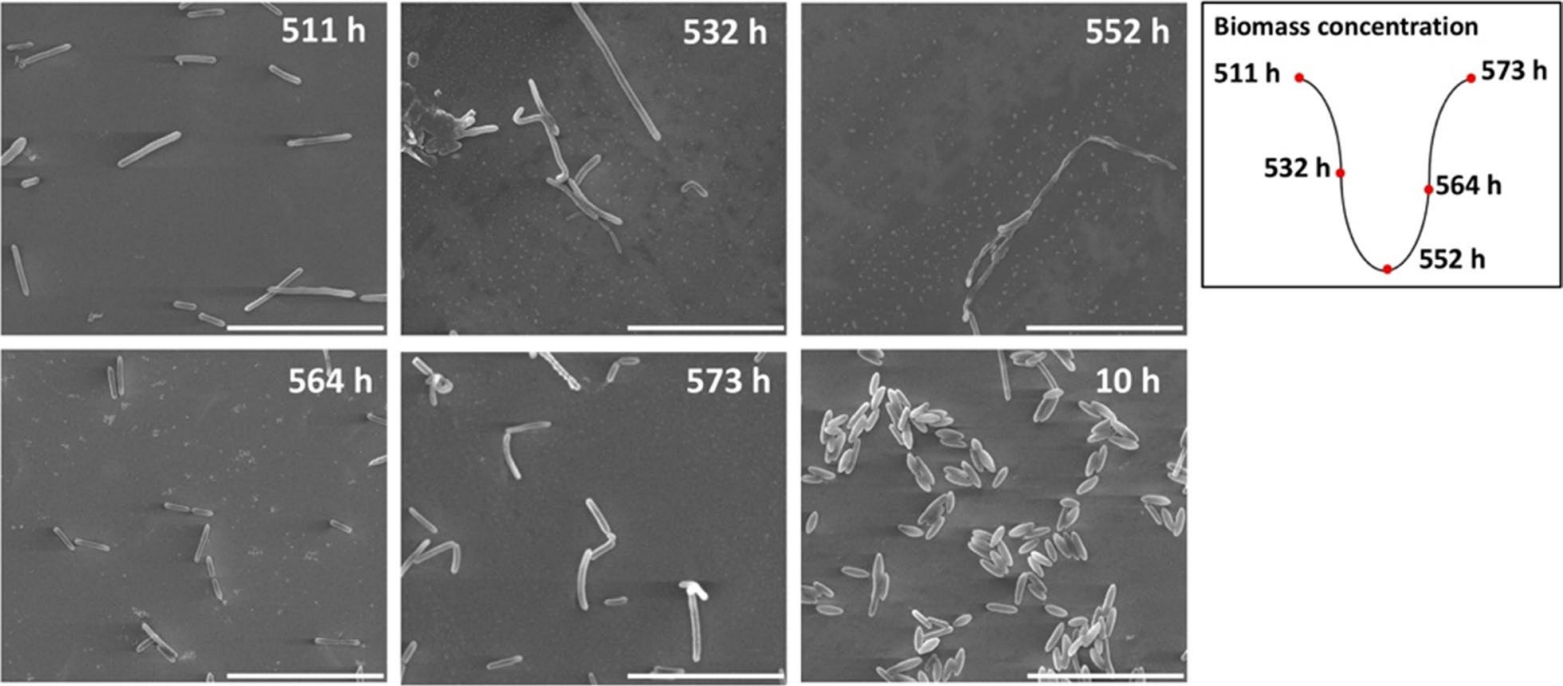
Changes in morphology of *C. butyricum* S3 during an oscillation cycle. Operating condition: continuous fermentation at a glycerol feed concentration of 88 g/L and a dilution rate of 0.048 h^−1^. Bar, 20 μm

*Clostridium butyricum* cells were harvested at five time points during one period of oscillation (511–573 h) as well as at initial stage (10 h) to track the morphological changes during long-term cultivation (Fig. 5). Overall, long-term operation resulted in significant changes of cellular morphology, as microbial cells showed slimmer and more elongated rod shapes at 511–573 h compared with the homogeneous fusiform shapes at the early stage of the fermentation (10 h). During one period of oscillation (511–573 h), significant changes in cell morphology were observed. At 511 h when the biomass production achieved the maximum, microbial cells exhibited inhomogeneous filament shapes with various length scales from 1 to 12 μm. While after 21 h when the biomass concentration decreased to approximately half of the maximum value (532 h), the size distribution of the microbial cells became more inhomogeneous with length scale from 1 to 20 μm. Cell aggregates and multiple cell debris/spores were observed at this time. Afterwards, at 552 h with the lowest biomass concentration, the cell image was occupied by cell debris/spores with few elongated and aggregated cells. By 564 h when cells recovered to approximately half of the maximum biomass concentration with the highest growth rate, microbial cells exhibited the most homogeneous size distribution among the oscillation, with fewer fragments/spores in the image. Finally, at 573 h when biomass concentration increased to the maximum value, cell morphology returned to the conditions similar to that at 511 h, with nearly no fragments/spores but inhomogeneous free cells.

### Genome and transcriptome

For further analysis of the metabolic pattern of this newly isolated 1,3-PDO producer, the genome of *C. butyricum* S3 was sequenced and annotated. The total length of the genome is 4350028 bp with low G + C content of 28.56%, which is accordance with the feature of low G + C content in the genomes of *C. butyricum* [35]. The sequence was assembled into 214 scaffolds with N_50_ of 50728 bp. A total of 3738 genes were identified by NCBI Prokaryotic Genome Annotation Pipeline (PGAP). The genome information was used as the reference for transcriptome assembly.

**Fig. 6:**
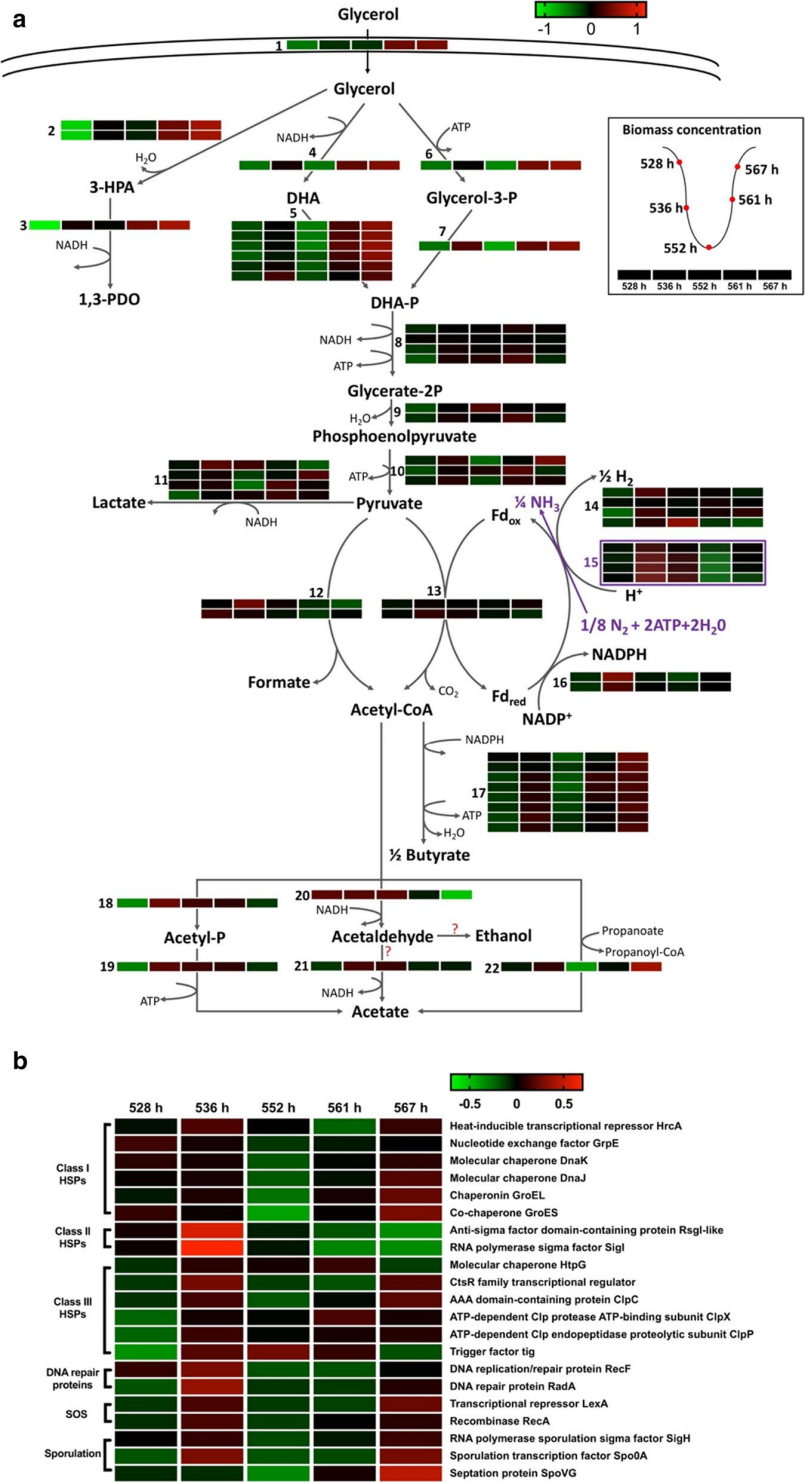
Expression levels of genes involved in glycerol metabolism (**a**) and stress response (**b**) for *C. butyricum* S3 at five time points (from left to right: 528 h, 536 h, 552 h, 561 h and 567 h) during an oscillation cycle. Operating condition: continuous fermentation at a glycerol feed concentration of 88 g/L and a dilution rate of 0.048 h^−1^

The transcriptomes of *C. butyricum* were analyzed in continuous fermentation at five time points during one oscillation cycle (528 h, 536 h, 552 h, 561 h and 567 h) (Additional file 2: Table S1). In this paper, genes involved in glycerol metabolism and stress response were paid close attention because of the dramatic changes of metabolic profile and morphology. Figure 6a (Additional file 2: Table S2) summarizes the expression profiles of the key genes related to glycerol metabolism. Genes with locus tag of GND98_RS15995 and GND98_RS16000 which were annotated initially as pyruvate formate lyase and its activator protein by NCBI PGAP as well as other databases, were predicted to encode glycerol dehydratase and its activator protein. Two evidences supported this hypothesis [13]: the similarity feature of these two proteins were accordance with that of the vitamin B12-independent glycerol dehydratase and its activating protein in *C. butyricum* VPI 1718; the gene (GND98_RS16005) next to the above genes was annotated as 1,3-PDO oxidoreductase (EC 1.1.1.202) by KO database (Additional file 2: Tables S1 and S2), which was consistent with the previous study that the three genes were in the same operon (*dha* operon).

In general, genes encoding glycerol uptake (pathway 1), glycerol reduction to 1,3-PDO (pathways 2–3) and glycerol oxidation to pyruvate (pathways 4–10) showed consistent expression patterns during one cycle of oscillation: the lowest expression occurred at stage II (528 h or 552 h), and the highest expression level at stage I (561 or 567 h), which was accordance with the metabolic profile. It was worthy attention that for 1,3-PDO production (pathway 2–3), three genes encoding glycerol dehydratase (GND98_RS15995) and its activator protein (GND98_RS16000), and 1,3-PDO oxidoreductase (GND98_RS16005) showed the most significant expression changes during an oscillation cycle, with 44-, 45- and 75-fold up-regulated at 567 h compared with that at 528 h, respectively. For glycerol oxidation, two parallel pathways for converting glycerol to dihydroxyacetone phosphate (DHA-P) were identified in *C. butyricum* S3: first, glycerol is converted to DHA by glycerol dehydrogenase (GND98_RS15960, pathway 4), then to DHA-P by dihydroxyacetone kinase (six genes listed in Additional file 2: Table S2, pathway 5). Second, glycerol is converted to glycerol-3-phosphate (glycerol-3-P) by glycerol kinase (GND98_RS14080, pathway 6) and then to DHA-P by glycerol-3-phosphate dehydrogenase (GND98_RS14075, pathway 8). All genes participating in these two pathways showed consistent expression patterns, with significantly lower level at 528 h and 552 h than at other stages and the highest level at 567 h.

For further degradation of pyruvate, three pathways existed in *C. butyricum* S3. First, for lactate production (pathway 11), four genes (GND98_RS11690, GND98_RS12540, GND98_RS12925, GND98_RS14955) were annotated as L-lactate dehydrogenase (LDH, EC 1.1.1.27), which showed inconsistent expression patterns during one cycle of oscillation (Fig. 6a, Additional file 2: Table S2). Alternatively, pyruvate could be converted to acetyl-CoA by pyruvate: formate lyase PFL (EC 2.3.1.54, pathway 12) and/or pyruvate: ferredoxin oxidoreductase PFO (EC 1.2.7.1, pathway 13) coupled with production of formate and reduced ferredoxin, respectively. For formate production, the genes coding PFL and PFL activating proteins showed a remarkably reversed expression pattern from that encoding enzymes for pathways 1–10, with higher expression levels at stage II (528–552 h) than that at stage I (561–567 h). Specifically, genes encoding PFL (GND98_RS14935) showed over two-fold higher expression level at 536 h than at other oscillatory stage. Gene encoding PFL activating protein (GND98_RS14940) showed the highest expression level at 528 h, 2.7-fold higher than the lowest level at 561 h. In contrast, expression level of the gene encoding PFO (GND98_RS01050, pathway 13) showed no significant changes during an oscillation cycle, although the average expression level was much higher than that for PFL, with average TPM level of 3404.29 vs. 409.97 during 528 to 567 h.

Next, for catalysis of the oxidation of reduced ferredoxin, according to previous studies [12, 36], two pathways existed in *C. butyricum*: via hydrogenase and/or via ferredoxin: NADH reductase. For hydrogenase, the gene with locus tag of GND98_RS03195 was 98.67% similar with gene encoding hydrogenase HydA in *C. acetobutylicum* DSM 792 (EF627973) [37]. This gene showed the highest expression level at middle of stage II at 536 h. Meanwhile, another two genes encoding hydrogenases hydG (GND98_RS14165) and hydF (GND98_RS19635) presented the highest expression levels at stage II as well, at 536 h and 552 h, respectively. That is, expression levels of the genes encoding all hydrogenases showed maximum at stage II instead of stage I, which was opposite to that of genes for 1,3-PDO production and glycerol oxidation to pyruvate. The results were accordant with the metabolic profile that hydrogen production lagged behind cell growth and 1,3-PDO production. In addition, four genes (GND98_RS11065, GND98_RS11070, GND98_RS11075, GND98_RS11080) encoding different subunits of nitrogenase (EC 1.18.6.1) were found highly expressed during an oscillation cycle (pathway 15, marked in purple). Nitrogenase can oxidize reduced ferredoxin using nitrogen as electron acceptor, accompanying with ATP consumption and H_2_ production [38]. Expression patterns of the genes were similar with that for hydrogenases, with the highest level at stage II (536 h), whereas the lowest level at stage I (561 h). Another pathway to regenerate ferredoxin is by ferredoxin-NAD^+^ reductase (EC 1.18.1.3). To our surprise, genes encoding this enzyme were not found in the genome of *C. butyricum* S3. Alternatively, two genes (GND98_RS12070, GND98_RS12075) encoding ferredoxin-NADP^+^ reductase (EC 1.18.1.2, pathway 16) that using NADP^+^ as the electron cofactor showed considerable expression levels. The genes expressed highest level at 536 h as well, which was consistent with that encoding hydrogenase and nitrogenase. Thus, all genes encoding enzymes for oxidation of reduced ferredoxin exhibited reverse expression patterns during one cycle of oscillation.

Acetyl-CoA is the branch point intermediate for further synthesis to main byproducts, i.e. of butyrate and acetate. For butyrate production (pathway 17), all relevant genes showed relatively consistent change patterns during an oscillation cycle with the highest expressions at stage I (567 h) and the lowest expressions at stage II (528/552 h), which was similar with that for pathway 1–10. For acetate production, however, both genes encoding phosphate acetyltransferase (EC 2.3.1.8, GND98_RS01915, pathway 18) and acetate kinase (EC 2.7.2.1, GND98_RS01920, reaction 19) were strongly up-regulated from 528 to 536 h (10.93- and 8.41-fold, respectively), following by gradually down-regulated from 536 to 567 h. This expression pattern was inconsistent with acetate kinetics, in which acetate production was completely suppressed during time 528–550 h, but highly activated during 561–567 h (Fig. 4). Besides, acetyl-CoA could be converted to acetaldehyde and potentially to ethanol by bifunctional acetaldehyde-CoA/alcohol dehydrogenase (EC1.2.1.10, 1.1.1.1). The gene (GND98_RS12095) was highly expressed during an oscillation cycle and showed significantly higher expression levels at stage II (528 h-552 h) than at stages I (561 h and 567 h) (17.41-fold up-regulated at 528 h compared to that at 567 h). This pattern was exactly opposite to that for pathway 1–10. Ethanol was not detected during the entire fermentation (data not shown). Alternatively, acetaldehyde could be further converted to acetate by aldehyde dehydrogenase (EC 1.2.1.3). Although the gene encoding this enzyme was found in *C. butyricum* S3 (GND98_RS02310), its average expression level was 235 times lower than that for bifunctional acetaldehyde-CoA/alcohol dehydrogenase (average TPM value of 6.93 vs. 1631.99). Thus, there was no sufficient evidence to suggest that acetaldehyde was further converted to acetate or accumulated during the fermentation. In addition, the gene (GND98_RS14095) encoding propionate CoA-transferase (EC 2.8.3.1) that is able to catalyze conversion of acetyl-CoA to acetate (pathway 22) was found in *C. butyricum* and exhibited similar expression tendency with that for pathways 1–10.

Oscillatory behavior could be treated as a response of stress relative to long-term substrate limitation. Heat shock proteins (HSPs) are molecular chaperones that help cells to repair and degrade proteins damaged by temperature, salt, solvent or other stresses [39]. Expression of HSPs during one cycle of oscillation are shown in Fig. 6b (Additional file 2: Table S2). Genes encoding class I HSPs GrpE, DhaK, DhaJ and GroES, GroEL, and their repressor HrcA [40] showed significantly higher expression level at 528, 536 or 567 h when cells showed decreasing specific growth rates, than that at 552–561 h when cells exhibited increasing specific growth rates (Fig. 4). For class II HSPs, genes encoding sigma factor SigI along with anti-sigma factor RsgI-like [41] were identified in the genome of *C. butyricum* S3 (GND98_RS03420, GND98_RS03415). Both genes were significantly up-regulated at 536 h compared with other stage of oscillation especially at 567 h (57- and 162-fold for *sigI* and *rsgI*, respectively). Furthermore, multiple genes encoding class III HSPs (HtpG, CstR, ClpC, ClpX, ClpP, Tig) were identified in *C. butyricum* S3 and expressed higher levels at 536 h. Genes encoding class IV HSPs (HtrA) were not identified in the genome of *C. butyricum* S3, but two genes encoding DNA repair proteins RadA (GND98_RS14600) and RecF (GND98_RS11345) were identified and expressed significantly higher levels at 536 h and 567 h than other oscillatory stages. Furthermore, LexA repressor and RecA activator are two core proteins involved in SOS response for microbial cells [42]. Both genes showed higher expression levels at 536 h and 567 h than at other stage. As bacterial SOS response is a global response to DNA damage [42], the expression trends of the SOS-related genes indicated that during oscillations, microbial cells may suffer from dramatic DNA damage. For sporulation, many genes involved in spore formation were identified in the genome of *C. butyricum* S3 (Additional file 2: Table S2). Three of them showed significantly higher expression levels than others during one oscillation period (highlight in red in Additional file 2: Table S2): genes encoding RNA polymerase sporulation sigma factor SigH (GND98_RS14650), the master regulator for endospore formation Spo0A (GND98_RS02685) and septation protein SpoVG (GND98_RS02835). All the three genes showed significantly higher expression level at 536 h and 567 h than that at other oscillatory stage (Fig. 6b).

## Discussion

*C. butyricum* is a promising candidate for production of various biochemicals as well as an important resident in human intestinal [43]. *C. butyricum* S3, a competitive 1,3-PDO producer used in our study, exhibited unstable performance in long-term continuous fermentation not by accident, but under all conditions whenever substrate glycerol feed is sufficient [19] or limited (this study). Thus, the metabolic shift is less likely caused by gene mutation or contamination [18], but more likely a response to certain stress derived from long-term continuous operation. In this study, the oscillatory behavior was chosen to explore the potential mechanism behind this physiology. To the best of our knowledge, this is the first report to systematically investigate the oscillatory behavior by *C. butyricum*.

Occurrence of oscillation by *C. butyricum* was bound to operating conditions. Combined with the previous study [19], spontaneous oscillations were only observed at low dilution rates, and the residual glycerol concentrations remained low before oscillation (Additional file 2: Table S3). Merely increasing the dilution rate would lead to disappearance of the oscillation by either *C. butyricum* S3 or *C. butyricum*-intensive microbial consortium C2-2M under various glycerol feed concentrations (88, 110 and 130 h). For example, in continuous fermentation of *C. butyricum* S3 at a glycerol feed concentration of 88 g/L, no oscillation was observed when increasing the dilution rate from 0.048 to 0.096 h^−1^, although most glycerol was consumed under this condition as well (Fig. 2b). Thus, it seems that the glycerol-limited condition is a prerequisite for occurrence of oscillatory behavior by *C. butyricum*. Meanwhile, when further limiting substrate supply by decreasing the glycerol feed concentration to 44 g/L, the oscillatory behavior could be triggered but not sustained at low dilution rates of 0.048 or 0.096 h^−1^ (Fig. 2c, d). The results indicated that besides glycerol limitation, a minimum concentration of glycerol in feed medium (in this study 88 g/L) is additionally required for *C. butyricum* S3 to maintain oscillation. Based on the results, one common reason for oscillation occurrence was ruled out: toxicity of the known end products [26], as in continuous fermentation with sufficient glycerol supply, no oscillation was observed despite comparable productions of 1,3-PDO, butyrate, acetate, lactate and formate [19].

The typical oscillatory behavior of *C. butyricum* S3 is very unique from that found in other prokaryotic microorganisms such as *K. pneumoniae*, *C. acetobutylicum* and *C. pasteurianum*. First, it was not triggered by the environmental perturbation [27, 44] but occurred spontaneously. Second, the continuous oscillation was not come out at the beginning of the cultivation like *C. pasteurianum* [26], but after a period over 100 h during which cells exhibited relative stable growth and metabolism (Fig. 1). Third, the oscillation of *C. butyricum* S3 was much greater than that of the above microorganisms. During oscillations, *C. butyricum* S3 may face a life-death situation with zero or even negative specific growth rates (Fig. 4), occurrence of abnormal cell aggregates and debris/spores (Fig. 5) as well as overexpressed stress-related genes (Fig. 6b) at the trough of the oscillation.

During oscillations, *C. butyricum* S3 exhibited an overall metabolic cycle, in which concentrations of glycerol and all metabolites exhibited periodic fluctuations (Fig. 1). Productions of lactate and formate lagged behind microbial growth and productions of other metabolites including 1,3-PDO and butyrate. Kinetic analysis showed that during one periodic cycle, two peaks occurred for specific production rates of lactate and formate. First peak occurred at middle of rising stage, which was accordant with that of other metabolites. Another peak occurred in the middle of falling stage when synthesis of other products was suppressed. As both lactate and formate are direct products from pyruvate metabolism (Fig. 6a, pathway 11 and 12, respectively). The abnormal patterns of these two acids directly pointed to disorder of pyruvate metabolism during oscillations. In *C. butyricum* species, pyruvate is mainly converted to acetyl-CoA by pathway 13 via pyruvate: ferredoxin oxidoreductase instead of pathways 11 and 12 [12]. In consideration of all factors, it is suspected that the abnormal patterns of lactate and formate productions are response to the blocked main pyruvate-degradation route (pathway 13), resulting in activation of alternative pathways when cells were under stress. In fact, lactate and formate are not the major byproducts in glycerol metabolism in species of *C. butyricum*. Lactate tended to accumulate when *C. butyricum* were under stress, for example in fed-batch fermentation using crude glycerol instead of pure glycerol as substrate [45], and in long-term continuous fermentations just before the performance degradation [19] or oscillation (this study). It is indicated that the glycerol metabolic shift to lactate accumulation might be the common phenomenon and first response of *C. butyricum* when under stress. In comparison, formate production was only observed in two *C. butyricum* species in continuous fermentation: VPI3266 [36] and S3 (this study) to the best of our knowledge. As *C. butyricum* S3 exhibited remarkable metabolic shift from lactate production to formate production at later period of oscillation (Fig. 1, Additional file 1: Figs. S1, S3, S4), it is suspected that activation of formate pathway is the second response of *C. butyricum* S3 after long-term continuous operation under glycerol-limited conditions. As for reasons for metabolic shift from lactate to formate production at later period of continuous fermentation, two hypotheses are proposed. First, *C. butyricum* S3 may undergo metabolic self-optimization in response to glycerol-limited condition. Although both lactate and formate are the products of pyruvate metabolism, lactate production requires NADH as the reducing power without production of ATP. In contrast, formate production is accompanied by formation of acetyl-CoA, and acetyl-CoA would further convert to byproducts such as butyrate and acetate to generate energy and reducing power (Fig. 6). After long-term glycerol-limited operation, the metabolic pathway shifted from lactate to formate production so as to generate more energy and reducing power, and support cell growth and 1,3-PDO production. Second, the significant metabolic shift may be caused by accumulation of certain toxic intermediates/products during long-term glycerol-limited operation.

Another noticeable phenomenon for metabolic profile is the changing pattern of H_2_ production. The production rate of H_2_ showed delayed pattern compared with that of cell growth and synthesis of main products represented by 1,3-PDO. For H_2_ production pathway, the possibility of H_2_ production from formate split was first excluded, as neither the gene encoding formate dehydrogenase existed in the genome nor detectable activity of the enzyme was observed (data not shown). Thus, H_2_ could only be produced by pathways 14 (hydrogenase) and 15 (nitrogenase), both of which were coupling with re-oxidization of reduced ferredoxin. Ferredoxin is the coenzyme of pyruvate: ferredoxin oxidoreductase for pyruvate metabolism. Again, the opposite tendency for H_2_ production indicated the disorder of pyruvate metabolism as well, specifically pointing at ferredoxin cycle.

Redox balance is critical for glycerol dissimilation, which is strongly bound up with the metabolic flux of microbial cells [46–48]. Extracellular redox potential would significantly affect the intracellular redox status and vice versa [49]. The consistent fluctuation of intracellular (NAD^+^/NADH ratio) and extracellular (ORP) redox status indicated that during oscillations, the microbial cells underwent distinct redox alternative from an oxidized state to a reduced state corresponding to the kinetic profiles of biomass and 1,3-PDO production. In addition, NAD^+^/NADH is believed as the primary redox couples for glycerol metabolism, as 1,3-PDO production required NADH as reducing power, which is produced via glycerol oxidation. But in this study, NADP^+^/NADPH participated in glycerol metabolism of *C. butyricum* especially for ferredoxin regeneration (pathway 16) as well. Changes in intracellular NADP^+^/NADPH levels during oscillation requires further exploration for comprehensive assessment of intracellular redox status. Furthermore, ORP value was at maximum when the specific growth rate was maximum, followed by instant decrease to the minimum as soon as the growth rate passed through the peak. Thus, ORP value could be the real-time monitor of cell condition and oscillatory behavior for future research.

Multiple studies showed that microbial cells would become elongated and form long filaments under different stressful conditions, which is a signal for population being over-stressed, sick and dying [50]. For example, *C. tyrobutyricum* became filaments/long chains of rods at low pH values (pH < 4.3), whereas the cells recovered to normal rods when cultured at pH 4.7 [51]. *C. butyricum* DSP1 showed similar elongated forms under osmotic stress conditions (170 g/L initial glycerol concentration) [52]. This filament-liked shapes indicated that microbial cells were under stressful conditions after long-term operation under glycerol-limited condition. It would be valuable to investigate the morphology changes during long-term operations with unstable performance for *C. butyricum* S3 [19], as morphology change may act as an indicator to evaluate the cell condition during industrial long-term continuous fermentation. Nevertheless, during one period of oscillation, significant periodic changes of cell morphology were observed, which was consistent with microbial growth kinetics: *C. butyricum* exhibited highly heterogeneous length distributions along with aggregates and cell debris/spores at 532 h and 552 h when cells were under decreasing rate of cell growth, whereas the most uniform appearance was shown at 564 h when the cell growth rate achieved maximum. Similar periodic morphological changes were observed in oscillatory behavior of *C. acetobutylicum*. This oscillation is caused by the periodic shift within the population proportions between acidogenic and solventogenic cells during continuous fermentation [30]. In this study, the oscillatory behavior may also be associated to different life stages of *C. butyricum* during cultivation. Despite no evidence to determine whether the particles shown at 532–564 h were cell debris or spores, there was no doubt that microbial cells suffered from tremendous all-around damages during the falling stage during oscillations including metabolism, redox and morphology.

From genome information of *C. butyricum* S3, several discoveries about glycerol metabolism attracted attentions. First, in *C. butyricum* S3, the glycerol dehydratase and its activator protein (gene locus tag: GND98_RS15995, GND98_RS16000) showed most similarity with pyruvate formate lyase and pyruvate formate lyases activating enzymes, respectively. Based on the previous study [13], the glycerol dehydratase in *C. butyricum* S3 is most likely vitamin B12-independent as that in *C. butyricum* VPI 1718. This feature makes *C. butyricum* S3 more competitive in industrial application, with no need of additional nutrient for vitamin B12 supplementary. Second, for oxidization of glycerol to DHA-P, two parallel pathways were found in *C. butyricum* S3 (pathways 4–5, 6–7) and the relevant genes involved in both metabolic pathways expressed considerable levels during one oscillation period (Additional file 2: Table S2). The result is different from the previous study for *C. butyricum* VPI 3266 which relies only on pathways 4–5 to achieve glycerol oxidization [36]. Third, for regeneration of ferredoxin, the key electron carrier protein for pyruvate metabolism, it is surprised that no gene encoding ferredoxin: NAD^+^ reductase was found, which was considered to be the key enzyme for ferredoxin regeneration for *C. butyricum* [12, 36]. Instead, genes encoding ferredoxin-NADP^+^ reductase that using NADP^+^ as the coenzyme for ferredoxin regeneration were found and showed periodic expression levels during one cycle of oscillation (pathway 16). This is the first time that this NADP^+^-dependent ferredoxin regeneration pathway has been reported to take part in glycerol metabolism in *C. butyricum*. Last, genes encoding nitrogenase that catalyzes the regeneration of ferredoxin along with H_2_ production and N_2_ fixation were found and expressed high levels during one oscillation period (pathway 15), which is also the first report that nitrogenase was involved in glycerol metabolism for *C. butyricum*.

Among all analyzed genes, in general, there are two opposite expression patterns during one oscillatory cycle (Fig. 6): type I with the highest levels at rising stage (561, 567 h) and lowest levels at falling stage (528–552 h), or type II vice versa. Transcriptional patterns for Type I genes were consistent with the growth and kinetic profile, including genes for glycerol uptake (pathway 1), glycerol reduction to 1,3-PDO (pathway 2–3), glycerol oxidation to pyruvate (pathway 4–10) and butyrate biosynthesis (pathway 17). While type II genes showed unexpectedly high expression level at falling stage when microbial cells were undergoing apoptosis, including genes encoding for formate production (pathway 12), ferredoxin oxidation via hydrogenase (pathway 14), nitrogenase (pathway 15) and ferredoxin-NADP^+^ reductase (pathway 16), part of acetyl-CoA degradation (pathway 20) as well as multiple stress-response-related genes including that encoding type II HSPs and DNA repair proteins. The reverse changing patterns of type II genes indicated that the related pathways were potentially the trigger or the response for oscillatory behavior. The transcript levels for Type II genes involved in glycerol metabolism were consistent with the metabolic profiles of formate and H_2_. For lactate pathway, further research is needed to identify the changing pattern of LDH activity during oscillations. Disorder of pyruvate metabolism during oscillations is further confirmed by transcriptome analysis, which may be relative to the block of ferredoxin cycle probably caused by long-term substrate limitation. It is surprised that gene encoding PFO (pathway 13) did not show periodic expression, and extensive research around this enzyme and relevant pathways is needed. Disorder of pyruvate metabolism is also the potential reason for oscillatory behavior of another 1,3-PDO producer *K. pneumoniae* [28]. However, the disorder of pyruvate metabolism in *K. pneumoniae* results from oscillation of enzyme activities of pyruvate dehydrogenase and pyruvate formate lyase, which is triggered by drastic perturbation of cultivation condition. The reason for oscillatory behavior of *C. butyricum* S3 is obviously different, as it occurs spontaneously, and gene encoding pyruvate dehydrogenase does not exist in the genome of *C. butyricum* S3.

Product feedback inhibition is one of the most common reasons for oscillatory behavior of microbial cells. In previous studies, the intermediate 3-HPA was detected with high levels during oscillation of *K. pneumoniae*, which is highly toxic to microbial cells [27]. In this study, the oscillatory behavior of *C. butyricum* S3 was also possibly caused by excessive accumulation of toxic product(s), in consideration of inhibition of global metabolism, abnormal morphology as well as high expression levels of genes related to stress response and spore formation during the falling stage. Combined with results that oscillation only occurred under glycerol-limited conditions, it is suspected that long-term lack of growth factors represented by glycerol may lead to metabolic shift of *C. butyricum* S3, accumulation of toxic intermediates/products and finally the oscillatory behavior. First, accumulation of intermediate 3-HPA was first excluded, as the concentrations were maintained constantly low during the entire oscillation (data not shown). Alternatively, acetaldehyde, another highly toxic intermediate that is produced via pathway 20, may be accumulated during oscillations. There are several experimental evidences to support this hypothesis: (1) expression level of the gene encoding bifunctional acetaldehyde-CoA/alcohol dehydrogenase (pathway 20) showed the most significant and reverse changes compared to that of other essential genes during an oscillation cycle; (2) ethanol was not detected during oscillations and significantly low expression of gene encoding the alternative pathway for acetaldehyde degradation (pathway 21); (3) acetate is considered to be produced via pathways 18–19 with ATP production instead of pathway 21 in *C. butyricum* according to previous studies [53, 54]; (4) as conversion of acetyl-CoA to acetaldehyde requires NADH as reducing power (pathway 20), acetaldehyde accumulation would lead to fluctuation of NAD^+^/NADH ratio, which was corresponding to the results in Additional file 1: Fig. S5. All these evidences indicated the acetaldehyde accumulation was possible during oscillations. Acetaldehyde is a highly toxic intermediate for prokaryotic and eukaryotic organisms as an inhibitor of a wide range of enzymatic reactions, which could be accumulated when cells were under stress conditions [55–57]. Further researches need to be done to identify the intracellular and extracellular acetaldehyde concentration. It is also possible that potential mechanism of these two oscillation-relative phenomena: disorder of pyruvate metabolism and excessive acetaldehyde accumulation are relative to each other.

## Conclusion

In this study, a newly found spontaneous oscillation of *C. butyricum* was characterized in continuous fermentation using glycerol as the substrate for 1,3-PDO production, in terms of occurrence conditions, macroscopic characteristics as well as molecular profiles. The oscillatory behavior seems to only occur under glycerol-limited conditions at low dilution rates. During one cycle of oscillation, metabolic and kinetic analysis showed that productions of lactate, formate and H_2_ were significantly lagged behind compared with that of other metabolites. SEM picture showed multiple aggregates and multiple cell debris/spores occurring at the trough of oscillation. Transcriptome analysis showed that expression levels of the genes encoding formate production, ferredoxin oxidation via hydrogenase, nitrogenase and ferredoxin-NADP^+^ reductase, part of acetyl-CoA degradation as well as multiple stress-response-related genes exhibited reverse patterns from the metabolic profiles. Based on the existing results, it is presumed that long-term substrate limitation triggered two oscillation-related phenomena in *C. butyricum* S3: intracellular pyruvate metabolism disorder and excessive accumulation of acetaldehyde.

## Methods

### Microorganisms and cultivation media

*C. butyricum* S3, isolated from anaerobic microbial consortium was used in this study [58]. Cells were stored in seed medium supplement with 20% glycerol at − 70 °C. Crude glycerol provided by Sichuan Tianyu Oleochemical Co., Ltd., China was used as substrate in this study [58]. The composition of seed medium per liter was: 28 g crude glycerol (22 g glycerol content), 1.3 g KH_2_PO_4_, 4.454 g K_2_HPO_4_·3H_2_O, 2 g (NH_4_)_2_SO_4_, 0.2 g MgSO_4_·7H_2_O, 1 g yeast powder, 2 g CaCO_3_, 0.005 g FeSO_4_·7H_2_O, 0.02 g CaCl_2_, 0.5 g L-cysteine·HCl, 2 mL trace element solution A (V_A_). V_A_ contained (per liter): 0.9 ml HCl (12 M), 0.02 g CuCl_2_·2H_2_O, 0.07 g ZnCl_2_, 0.1 g MnCl_2_·4H_2_O, 0.06 g H_3_BO_3_, 0.2 g CoCl_2_·6H_2_O, 0.025 g NiCl_2_·6H_2_O, 0.035 g Na_2_MoO_4_·2H_2_O. The composition of fermentation medium per liter was: 1.36 g KH_2_PO_4_, 6.61 g (NH_4_)_2_SO_4_, 0.26 g MgCl_2_·6H_2_O, 0.29 g CaCl_2_, 0.42 g citrate, 2 g yeast powder, 5 mL trace element solution B (V_B_). V_B_ contained (per liter): 0.68 g ZnCl_2_, 0.17 g MnCl_2_·4H_2_O, 0.06 g H_3_BO_3_, 0.47 g CuCl_2_·2H_2_O, 0.005 g Na_2_MoO_4_·2H_2_O, 3.97 g FeCl_2_·6H_2_O, 0.47 g CoCl_2_·6H_2_O, 10 mL HCl (12 M). The glycerol content varied according to the experimental design.

### Cultivation conditions

Seed cultures and continuous fermentations were performed as described previously (Zhou, et al. 2018). An oxidation–reduction potential (ORP) probe (Mettler-Toledo) was installed in the fermenter to monitor ORP online.

### Kinetic calculations

Specific growth rate (μ, h^−1^):

$${\mu=}\frac{1}{{\text{X}}}\cdot\frac{\text{dX}}{{\text{dt}}}+ \text{D} .$$

Specific rate of glycerol consumption (${\text{q}}_{\text{s}}$, g/(g·h)):

$${\text{q}}_{{\text{s}}} = \frac{1}{{\text{X}}}\left( {{\text{D}} \cdot {\text{S}}_{{\text{f}}} - \frac{{{\text{dX}}}}{{{\text{dt}}}} \cdot {\text{C}}_{{\text{S}}} - \frac{{{\text{dC}}_{{\text{S}}} }}{{{\text{dt}}}}} \right).$$

Specific rate of liquid end-product production (${\text{q}}_{\text{P}}$, g/(g·h)):

$${\text{q}}_{{\text{P}}} = \frac{1}{{\text{X}}}\left( {\frac{{{\text{dC}}_{{\text{P}}} }}{{{\text{dt}}}}{\text{ + D}} \cdot {\text{C}}_{{\text{P}}} } \right).$$

Specific rate of gas production (${\text{q}}_{{\text{H}}_{2}\text{/}{\text{CO}}_{2}}$, mmol/(g·h)):

$${\text{q}}_{{{\text{H}}_{2} {\text{/CO}}_{2} }} = \frac{1}{{\text{X}}}\left( {\frac{{{\text{dC}}_{{{\text{H}}_{2} {\text{/CO}}_{2} }} }}{{{\text{dt}}}}{\text{ + D}}_{{\text{G}}} \cdot {\text{C}}_{{{\text{H}}_{2} {\text{/CO}}_{2} }} } \right),$$

where X is the biomass concentration (g/L), D is the dilution rate (h^−1^), S_f_ is the glycerol feed concentration (g/L), C_S_ is the residual glycerol concentration in the reactor (g/L), C_P_ is the liquid product concentration, ${\text{C}}_{{\text{H}}_{2}\text{/}{\text{CO}}_{2}}$ is H_2_ or CO_2_ concentration (mmol/L), and D_G_ is the gas-phase dilution rate (h^−1^).

### Enzyme assays

Culture samples (14 mL) for enzyme assays were taken from the fermenter quickly to 15 mL chilling serum bottles. After distributing into the centrifuge tube anaerobically, cells were centrifuged at 12,000 rpm at 4 °C for 5 min and then washed with Tris buffer (50 mM Tris/HCl, 2 mM DTT, 0.1 mM MnSO_4_, pH 7.4) three times. Afterwards, the resuspended cells were sonicated five times for 20 s with 60 s intervals at a power of 200 W in an ice water jacket. Cell debris was removed by centrifugation at 12,000 rpm at 4 °C for 15 min. At each step, extracts were maintained under anaerobic conditions. Protein concentrations of cell extracts were determined according to Lowry et al. (1951) [59].

Specific activity of formate dehydrogenase (FDH) was analyzed according to Balzer et al. with minor modifications [60]: the reaction mixture (1 mL) contained 1.67 mM NAD, 167 mM sodium formate, 100 mM β-mercaptoethanol and 10 mM sodium phosphate buffer (pH 7.5). The reaction was initiated by adding 200 μL cell-free extract. The slope of absorbance increase at 340 nm at 30 °C was analyzed by Jasco V-560 UV spectrophotometer (Jasco, Tokyo, Japan). One unit of the enzyme activity was defined as the amount of enzyme that produced 1 mmol of NADH per minute at 30 °C.

### Determination of NAD^+^ and NADH pools

NAD^+^ and NADH were extracted and the concentrations were measured according to the previous study [61]. For extraction of NAD^+^ and NADH, 1 mL culture was first centrifuged at 12,000*g* at 4 °C for 5 min. Intracellular NAD^+^ and NADH was extracted from the cell pellets with 0.3 mL 0.2 M HCl and 0.3 mL 0.2 M NaOH at 50 °C for 10 min and then neutralized by 0.3 mL 0.1 M NaOH and 0.3 mL 0.1 M HCl in the iced bath, respectively. The cellular debris were removed by centrifuged at 12,000*g* at 4 °C for 15 min. Supernatants were transferred to new tubes and stored at −20 °C for the following NAD^+^ and NADH content determination.

NAD^+^ and NADH were determined by spectrophotometric enzymatic cycling assay. The assay mixture contained 0.12 mL reagent mixture (equal volumes of 0.1 M bicine buffer pH 8.0, 40 mM EDTA pH 8.0, ethanol, 14.2 mM 3-[4,5-dimethylthiazol-2-yl]-2,5-diphenyltetrazolium bromide (thiazolyl blue) and twice volumes of 16.6 mM phenazine ethosulfate), 0.06 mL H_2_O, and 0.01 mL extracted sample. The reaction was started by addition of 0.01 mL alcohol dehydrogenase (150 U/mL in 0.1 M bicine buffer pH 8.0). The rate of increase in absorption at 570 nm $\Delta$ A_570_ was measured by Varioskan Flash (Thermo Fisher Scientific Inc). Concentration of the NAD^+^ and NADH were determined by calibrating the slope ($\Delta$ A_570_/min) with that obtained using a series of standard solutions of NAD^+^ and NADH (0.01–0.05 mM) as reagents.

### Analytics

Concentrations of biomass, glycerol, 1,3-PDO, butyrate, acetate, lactate and formate were determined as previously described [19]. Ethanol concentration was determined by gas chromatography (GC) [62]. Productions of H_2_ and CO_2_ were determined by GC using a thermal conductivity detector (Techcomp, 7900). 3-HPA concentration was determined as previously described [27].

### Microscopy

Cells under different phase of oscillation were harvested and washed by 0.1 M phosphate buffer (pH 7.4) three times and fixed by 2.5% glutaraldehyde overnight at 4 °C. Then cells were washed by 0.1 M phosphate buffer (pH 7.4). After washed, the cells were dehydrated with ethanol solutions with increasing concentration (50%, 70%, 80%, 90%, 100% twice). After dehydration, the cell pellets were freeze-dried, sputtered with gold and observed by scanning electron microscope (SEM, FEI quanta 450).

### Genome sequencing and transcriptome analysis

For genome sequencing*, C. butyricum* were cultivated anaerobically in the seed media at 37 °C for 12 h. Cells (5 mL) were collected by centrifugation at 12,000 rpm for 10 min at 4 °C and the genome DNA were extracted immediately (TaKaRa Bio). The genome of *C. butyricum* S3 was sequenced on Illumina HiSeq × 10 platform with a 2*150 bp paired-end module (Majorbio, Shanghai, China). Raw data were filtered by Trimmomatic. Gene assembly using SOAPdenovo2, in which 214 scaffolds were obtained. The genes were announced by NCBI Prokaryotic Genome Annotation Pipeline (PGAP). The whole-genome shotgun project has been deposited at DDBJ/EMBL/GenBank under the accession WOFV00000000.2.

For transcriptome analysis, 50 mL cultures from five time points (528 h, 536 h, 552 h, 561 h and 567 h) were immediately removed from the fermenter and centrifuged at 12,000 rpm for 10 min at 4 °C in the continuous fermentation at a glycerol feed concentration of 88 g/L and a dilution rate of 0.048 h^−1^. After the centrifugation, the supernatant was discarded and the cell pellets were immediately frozen in liquid nitrogen and then kept at -70 °C until further extraction. Total RNAs were extracted by TRIzol reagent (Invitrogen). The rRNA was then removed by Ribo-Zero rRNA Removal Kit (Epicentre, San Diego, CA). For cDNA library construction, the rRNA-free mRNAs were fragmented and reversely transcribed into double-stranded cDNA. The cDNA was sequenced by Illumina Hiseq (Majorbio, Shanghai, China). All identified genes were further annotated by Clusters of Orthologous Groups of proteins (COG), Pfam, GO, NCBI non-redundant protein sequences (NR), Swiss-Prot and Kyoto Encyclopedia of Genes and Genomes (KEGG) databases. The gene expression levels were determined as Transcripts Per Million (TPM) [63]. For significance analysis, the read counts were normalized by trimmed mean of M values (TMM). Then, the significance of differentially expressed genes was analyzed by DEGseq with p-adjust < 0.001 and |log_2_(fold change)|≥ 1. The raw data were deposited into Sequence Read Archive (SRA) database under project number of PRJNA605467 and accession SRR11043383-SRR11043387.

## Supplementary information

**Additional file 1.** **Fig. S1.** Productions of biomass and organic acids of *C. butyricum* S3 in continuous fermentation at a glycerol feed concentration of 88 g/L and a stepwise decreasing dilution rate from 0.144 h^-1^ to 0.048 h^-1^.
**Fig. S2.** Productions of biomass and organic acids of *C. butyricum* S3 in continuous fermentation at a glycerol feed concentration of 88 g/L and a dilution rate of 0.096 h^-1^.
**Fig. S3.** Productions of biomass and organic acids of *C. butyricum* S3 in continuous fermentation at a glycerol feed concentration of 44 g/L and a dilution rate of 0.048 h^-1^.
**Fig. S4.** Productions of biomass and organic acids of *C. butyricum* S3 in continuous fermentation at a glycerol feed concentration of 44 g/l and a dilution rate of 0.096 h-1.
**Fig. S5.** Intracellular and extracellular redox status for *C. butyricum* S3 in continuous fermentation at a glycerol feed concentration of 88 g/L and a dilution rate of 0.048 h^-1^. (**a**) Intracellular concentrations of NAD^+^ and NADH; (**b**) the ratio of NAD^+^/NADH; (**c**) extracellular oxidation-reduction potential (ORP).

**Additional file 2.** **Table S1.** The complete transcriptome analyses of *C. butyricum* S3 during an oscillation cycle.
**Table S2.** Summary of expression levels of genes involved in glycerol metabolism and stress response during an oscillation cycle.
**Table S3.** Residual glycerol concentrations and metabolic behaviors (damped oscillation/sustained oscillation/no oscillation) of *C. butyricum* S3 or *C. butyricum*-intensive microbial consortium C2-2M in continuous fermentations under different conditions.

## Data Availability

The datasets supporting the conclusions of this article are included within the article and its additional files.

## Abbreviations

1,3-PDO: 1,3-Propanediol
PTT: Polytrimethylene terephthalate
3-HPA: 3-Hydroxypropionaldehyde
GDHt: Glycerol Dehydratase
PDOR: 1,3-Propanediol Oxidoreductase
GDH: Glycerol Dehydrogenase
LDH: Lactate Dehydrogenase
PFO: Pyruvate: Ferredoxin Oxidoreductase
PFL: Pyruvate: Formate Lyase
ORP: Oxidation–Reduction Potential
PGAP: Prokaryotic Genome Annotation Pipeline
DHA: Dihydroxyacetone
DHA-P: Dihydroxyacetone Phosphate
Glycerol-3-P: Glycerol-3-Phosphate
TPM: Transcripts Per Million
HSPs: Heat Shock Proteins
SEM: Scanning Electron Microscope
