# Supplementary material for: Metabolism, morphology and transcriptome analysis of oscillatory behavior of Clostridium butyricum during long-term continuous fermentation for 1,3-propanediol production
Source: Biotechnol Biofuels. 2020 Nov 25;13:191. doi: 10.1186/s13068-020-01831-8 (PMC7690194; doi:10.1186/s13068-020-01831-8)
Supplement: Supplementary file 1 — Additional file 1. Fig. S1. Productions of biomass and organic acids of C. butyricum S3 in continuous fermentation at a glycerol feed concentration of 88 g/L and a stepwise decreasing dilution rate from 0.144 h-1 to 0.048 h-1. Fig. S2. Productions of biomass and organic acids of C. butyricum S3 in continuous fermentation at a glycerol feed concentration of 88 g/L and a dilution rate of 0.096 h-1. Fig. S3. Productions of biomass and organic acids of C. butyricum S3 in continuous fermentation at a glycerol feed concentration of 44 g/L and a dilution rate of 0.048 h-1. Fig. S4. Productions of biomass and organic acids of C. butyricum S3 in continuous fermentation at a glycerol feed concentration of 44 g/l and a dilution rate of 0.096 h-1. Fig. S5. Intracellular and extracellular redox status for C. butyricum S3 in continuous fermentation at a glycerol feed concentration of 88 g/L and a dilution rate of 0.048 h-1. (a) Intracellular concentrations of NAD+ and NADH; (b) the ratio of NAD+/NADH; (c) extracellular oxidation-reduction potential (ORP). [file 13068_2020_1831_MOESM1_ESM.docx]

Fig. S1 Productions of biomass and organic acids of *C. butyricum* S3 in continuous fermentation at a glycerol feed concentration of 88 g/L and a stepwise decreasing dilution rate from 0.144 h^-1^ to 0.048 h^-1^

Fig. S2 Productions of biomass and organic acids of *C. butyricum* S3 in continuous fermentation at a glycerol feed concentration of 88 g/L and a dilution rate of 0.096 h^-1^

Fig. S3 Productions of biomass and organic acids of *C. butyricum* S3 in continuous fermentation at a glycerol feed concentration of 44 g/L and a dilution rate of 0.048 h^-1^

Fig. S4 Productions of biomass and organic acids of *C. butyricum* S3 in continuous fermentation at a glycerol feed concentration of 44 g/l and a dilution rate of 0.096 h^-1^

Fig. S5 Intracellular and extracellular redox status for *C. butyricum* S3 in continuous fermentation at a glycerol feed concentration of 88 g/L and a dilution rate of 0.048 h^-1^. (a) Intracellular concentrations of NAD^+^ and NADH; (b) the ratio of NAD^+^/NADH; (c) extracellular oxidation-reduction potential (ORP)
